# Association of intensive care unit delirium with sleep disturbance and functional disability after critical illness: an observational cohort study

**DOI:** 10.1186/s13613-018-0408-4

**Published:** 2018-05-08

**Authors:** Marcus T. Altman, Melissa P. Knauert, Terrence E. Murphy, Amy M. Ahasic, Zeeshan Chauhan, Margaret A. Pisani

**Affiliations:** 1 Yale University School of Medicine, 300 Cedar Street, P.O. Box 208057, New Haven, CT USA; 2 Section of Pulmonary, Critical Care and Sleep Medicine, Yale University School of Medicine, New Haven, CT USA; 3 Geriatrics, Yale University School of Medicine, New Haven, CT USA; 4 Department of Biostatistics, Yale School of Public Health, New Haven, CT USA; 5 Section of Pulmonary and Critical Care Medicine, Norwalk Hospital, Norwalk, CT USA; 6 Department of Internal Medicine, John T. Mather Memorial Hospital, Port Jefferson, NY USA

**Keywords:** Sleep disturbance, Disability, Activities of daily living, Delirium, Critical care, Post-ICU, Post-hospital

## Abstract

**Background:**

In medical intensive care unit (MICU) patients, the predictors of post-discharge sleep disturbance and functional disability are poorly understood. ICU delirium is a risk factor with a plausible link to sleep disturbance and disability. This study evaluated the prevalence of self-reported post-ICU sleep disturbance and increased functional disability, and their association with MICU delirium and other ICU factors.

**Methods:**

This was an observational cohort study of MICU patients enrolled in a biorepository and assessed upon MICU admission by demographics, comorbidities, and baseline characteristics. Delirium was assessed daily using the Confusion Assessment Method for the ICU. Telephone follow-up interview instruments occurred after hospital discharge and included the Pittsburgh Sleep Quality Index (PSQI), and basic and instrumental activities of daily living (BADLs, IADLs) for disability. We define sleep disturbance as a PSQI score > 5 and increased disability as an increase in composite BADL/IADL score at follow-up relative to baseline. Multivariable regression modeled the associations of delirium and other MICU factors on follow-up PSQI scores and change in disability scores.

**Results:**

PSQI and BADL/IADL instruments were completed by 112 and 122 participants, respectively, at mean 147 days after hospital discharge. Of those surveyed, 63% had sleep disturbance by PSQI criteria, and 37% had increased disability by BADL/IADL scores compared to their pre-MICU baseline. Total days of MICU delirium (*p* = 0.013), younger age (*p* = 0.013), and preexisting depression (*p* = 0.025) were significantly associated with higher PSQI scores at follow-up. Lower baseline disability (*p* < 0.001), older age (*p* = 0.048), and less time to follow-up (*p* = 0.024) were significantly associated with worsening post-ICU disability, while the occurrence of MICU delirium showed a trend toward association (*p* = 0.077).

**Conclusions:**

After adjusting for important covariates, total days of MICU delirium were significantly associated with increased post-discharge sleep disturbance. Delirium incidence showed a trend toward association with increased functional disability in the year following discharge.

## Background

Critical illness encompasses a diverse group of diseases that confer a high risk of both mortality and morbidity [1, 2]. Impairment and morbidity after critical illness are believed to be particularly high and likely include worsening of medical comorbidities, decreased quality of life, and emotional and physical pain for patients. Understanding the extent of poor functional outcomes and their predictors after intensive care unit (ICU) admission is therefore clinically meaningful.

Sleep disturbance has elicited attention as one important outcome of critical illness. Sleep is highly deranged in the ICU, where patients regularly experience poor sleep quality, sleep fragmentation, and reduced sleep efficiency [3–8]. Abnormal sleep may predispose patients to adverse medical outcomes of critical illness [9–11], as well as poor sleep following hospital discharge. A systematic review from our group found that the prevalence of self-reported sleep disturbance in post-ICU patients was 50–66.7% in the first month, and as high as 61% more than 6 months after discharge [12]. Similarly, polysomnography studies in post-ICU patients have shown abnormal sleep parameters and sleep architecture that normalize by 6-month follow-up [12–16]. These studies, however, have found few ICU factors associated with post-ICU sleep disturbance; those identified to date include number of chronic diseases, ICU severity of illness, substantial hospital acute stress symptoms, and hospital opiate use [17–19].

Another post-ICU outcome of importance is functional disability, in which physiologic insults from critical illness cause persistent disability in meeting the physical or social demands of the patient’s life [20, 21]. This could include deficits in basic activities of daily living (BADLs) such as eating or dressing, or in instrumental activities of daily living (IADLs) such as managing finances or taking medications. A systematic review of IADLs in critical illness survivors found that patients in 69% of studies had new or worse IADL dependencies after critical illness [22], and studies specifically in older adults have found that post-ICU disability is often highly prevalent [20]. Risk factors identified for disability in critical illness survivors are diverse and include older age, depression, ICU severity of illness, mechanical ventilation, and pre-ICU sensory impairment [22–24].

ICU delirium is a candidate risk factor with a plausible link to post-ICU impairment such as sleep disturbance and disability. Delirium is highly prevalent in the ICU. A recent meta-analysis estimated the prevalence in critically ill patients to be 31.8% [9, 25]. The etiology of delirium likely includes contributions of chronic medical comorbidity and cognitive impairment, sensory impairment, sedative/hypnotic use, and potentially sleep disturbance [9, 25, 26]. Overall, ICU delirium is associated with increased ICU length of stay, longer mechanical ventilation duration, and increased mortality [9, 25].

To our knowledge, no studies have assessed the influence of ICU delirium on sleep outcomes after critical illness, and its effects on post-hospital disability have been equivocal [24, 27, 28]. Overall, given the importance of sleep and functional recovery after critical illness, and the significant effect of delirium on other ICU outcomes, our aim was to evaluate both (1) the prevalence of self-reported sleep disturbance and increased disability post-ICU, and (2) the relationship between ICU delirium and post-ICU sleep disturbance and disability. Our secondary aim was to examine whether other previously studied ICU factors (such as severity of illness, length of stay, and mechanical ventilation) were related to these post-ICU outcomes.

## Methods

### Patient sample

We conducted an observational cohort study of critically ill adults admitted to the medical ICU (MICU) of a large academic tertiary medical center. The cohort was nested in a MICU biorepository in which critically ill patients with an expected ICU length of stay > 24 h were eligible for enrollment at time of MICU admission. Patients or appropriate surrogates completed admission interviews that included demographics and baseline functional status (BADLs and IADLs). Preexisting medical comorbidities and daily mechanical ventilation status were extracted from chart review. All patients were asked to provide contact information for future telephone follow-up. This study was approved by the Yale Institutional Review Board (IRB Number 1110009161).

### Delirium assessment in the ICU

MICU delirium was assessed daily during the first week of admission by trained research staff using the Confusion Assessment Method for the ICU (CAM-ICU) [29]. Using criteria previously validated in both ICU and non-ICU patients, research staff also performed daily patient chart review for evidence of delirium or acute confusion episodes by assessing flow sheets and physician and nursing notes [30, 31]. This involved chart abstraction for CAM-ICU documentation and evidence of key terms and descriptors suggestive of an acute confusional state, with coding based on the question “Is there any evidence from the chart of acute confusional state (for example, delirium, mental status change, inattention, disorientation, hallucinations, agitation, inappropriate behavior, or other)?” [30, 31]. Patients were considered to be delirium positive for the day if either assessment method was positive.

### Follow-up assessment

From August 2014 to May 2015 and November 2016 to February 2017, all eligible patients not known to be deceased were contacted for follow-up. Interviews were targeted for 3 months to 1 year after hospital discharge. Patients were called at varying times of day and across all days of the week. At least three attempts were made to contact patients, after which they were considered “unable to be contacted.” Patients were excluded from follow-up if their MICU stay was < 24 h, if they had previously been enrolled in the biorepository in a prior admission, or if their interview was completed > 365 days after hospital discharge.

### Follow-up instruments

Sleep quality was assessed using the Pittsburgh Sleep Quality Index (PSQI)—a validated 19-item instrument with questions spanning multiple sleep domains including sleep latency, sleep duration, sleep efficiency, and sleep disturbance [32, 33]. Domain scores are summed to a “global” count score from 0 to 21, with higher scores indicating worse disturbance, and scores > 5 being sensitive and specific for poor sleep quality [33]. We define sleep disturbance as having a PSQI score > 5. Patients with evidence of severe cognitive or hearing/language impairment limiting the interview were not assessed with this tool. Written permission was obtained to use this instrument.

Disability assessment at baseline and follow-up included seven BADLs (bathing, grooming, getting from bed to a chair, walking across a small room, dressing, eating, and toileting) and seven IADLs (using the telephone, shopping, preparing meals, household chores, taking medications, managing money, driving/navigating to places out of walking distance) [34, 35]. Because each item was scored as 0 or 1 (0, performs the activity unassisted; 1, needs some help or completely dependent), potential total disability scores ranged from 0 to 14, with higher scores indicating increased disability. For patients with cognitive or hearing/language impairment limiting the interview, disability information was obtained from a surrogate who had frequent contact with the patient. A previous study has suggested a high patient-proxy inter-observer agreement in assessing functional status in critical illness survivors [36]. Overall, we define increased disability as an increase in composite BADL/IADL score at follow-up relative to baseline.

During patient telephone interviews, PSQI questions were prioritized first, with BADL/IADL questions asked second if the patient was willing to continue. Surrogates were contacted if the patient was unable or unwilling to complete the interview.

### Statistical analysis

The prevalence of sleep disturbance at follow-up was calculated using the proportion of patients with PSQI scores > 5. The prevalence of increasing disability post-ICU was calculated using the proportion of patients who had increased composite ADL/IADL scores at follow-up relative to their admission interview.

The count outcome of total PSQI score was modeled using negative binomial regression. Explanatory variables included age, gender, baseline depression, Acute Physiology and Chronic Health Evaluation (APACHE) II score, hospital length of stay, mechanical ventilation status, time to follow-up interview, and total days of MICU delirium in the first week of ICU stay. An analogous model was also run instead using a binary indicator of MICU delirium (i.e., whether the patient was delirious for at least 1 day).

The change in disability count outcome between follow-up and baseline was modeled using linear regression. As with the PSQI scores, two models were run with the same explanatory variables—one using total days of MICU delirium and the other a binary indicator of occurrence of delirium for at least 1 day. Count of baseline disability was also included in the models of disability.

Associations for PSQI scores are reported as rate ratios, and associations for disability are reported as point estimates that represent the average amount in which the outcome changes per incremental change in the explanatory variables. For negative binomial regression (PSQI), the change in outcome is proportional, while for linear regression (disability) it is additive. In all cases, significance was defined as a two-sided *p* value < 0.05. Analyses were performed using SAS statistical software V9.4 (Cary, NC).

## Results

### Patient cohort development and characteristics

**Fig. 1:**
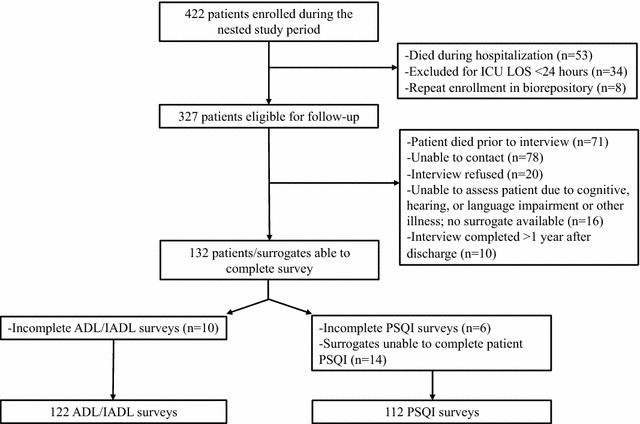
Consort diagram for cohort development, *ADL* activities of daily living, *IADL* instrumental activities of daily living, *ICU* intensive care unit, *LOS* length of stay, *PSQI* Pittsburgh Sleep Quality Index

From the MICU biorepository, 422 patients were enrolled during the study period, of which *n* = 327 were eligible for potential follow-up (Fig. 1). Of these, 22% died after hospitalization, but before follow-up, 24% were unable to be contacted, 5% were unable to participate due to cognitive or hearing impairment without available surrogate, and 6% refused interview. Ten patients (3%) were excluded because their interview was completed > 1 year after discharge. Overall, after accounting for incomplete surveys, 112 patients completed the full PSQI instrument within 1 year of hospital discharge (mean 145 days, SD 70). Disability questions were completed for 122 patients, with 11% (*n* = 14) of these being obtained from surrogates (mean 147 days, SD 69).

Of the 122 patients completing the disability surveys, the mean age was 65 years (SD 16), 50% were male, and 88% were Caucasian (Table 1). Thirty-six percent of patients had at least one day of MICU delirium (mean total days delirious = 2.9). The overall one-year mortality of this cohort of ICU survivors was 8.2% (*n* = 10; an additional 6.6% of patients had no one-year follow-up status available).

**Table 1:** Characteristics of patient population (*N* = 122)

Characteristic	Mean (SD) or %
Age in years	65.5 (15.6)
Gender	50.0% male
Race	87.7% white/Caucasian
APACHEII	18.8 (5.3)
Primary reason for ICU admission	
Acute respiratory failure/pulmonary edema	33.6%
Pulmonary embolism	4.1%
Sepsis/shock	28.7%
Pneumonia	3.3%
Acute kidney failure	2.5%
Gastrointestinal bleed	12.3%
DKA/HHS	2.5%
Severe metabolic derangement	5.7%
Alcohol withdrawal/drug overdose	2.4%
Other	4.9%
Infection	36.1%
Respiratory distress	45.9%
Medications in ICU*	
Opioids	56 (46%)
Benzodiazepines	47 (39%)
Propofol	16 (13%)
Delirium Source	
CAM by research staff	3%
Chart review	82%
CAM and chart review	15%
ICU LOS in days	4.2 (3.4)
Hospital LOS in days	10.4 (8.9)
% Mechanically ventilated	15.6%
Delirium: % of patients with delirium	36%
Delirium: Mean total days of delirium	2.9 (3.4)

*APACHE II* Acute Physiology and Chronic Health Evaluation II, *CAM* Confusion Assessment Method, *DKA/HHS* diabetic ketoacidosis/hyperosmolar hyperglycemic syndrome, *ICU* intensive care unit, *LOS* length of stay

*Opioids refers to receipt of fentanyl, morphine, or hydromorphone; benzodiazepines refers to receipt of lorazepam or midazolam

### Sleep disturbance at follow-up

The prevalence of poor sleep quality was 63% (*n* = 71) as defined by a PSQI score of > 5, and the mean PSQI score at follow-up was 8.58 (SD 5.17). Of the patients who completed the PSQI, 37% (*n* = 41) endorsed taking medication (either prescribed or over-the-counter) to help sleep in the past month, with 28% (*n* = 31) taking such medications at least three times per week.

Table 2 shows the results of negative binomial regression for factors associated with follow-up PSQI scores. Increasing days of MICU delirium were significantly associated with higher PSQI scores (*p* = 0.013). Increasing age was associated with lower PSQI scores (*p* = 0.013). Preexisting depression by chart review was associated with higher PSQI scores (*p* = 0.025). The other factors in the regression model were not associated with follow-up PSQI scores. Notably, when delirium was included as a binary variable (i.e., whether the patient was delirious for at least one day), similar results were obtained—age (*p* = 0.011), depression (*p* = 0.007), and occurrence of delirium (*p* = 0.003) were significantly associated with PSQI counts.

**Table 2:** Negative binomial analysis for factors associated with follow-up PSQI scores (*n* = 112)

Factor	Rate ratio (95% confidence interval)	*p* value
Age	0.990 (0.982, 0.998)	0.013*
Female gender	1.056 (0.851, 1.309)	0.620
Preexisting depression	1.335 (1.036, 1.720)	0.025*
APACHE II	1.005 (0.982, 1.028)	0.664
Hospital LOS	1.003 (0.993, 1.014)	0.531
Mechanically ventilated during ICU admission	0.867 (0.622, 1.208)	0.399
Time to interview in days	1.000 (0.998, 1.001)	0.541
Total days of delirium	1.114 (1.023, 1.212)	0.013*

*APACHE II* Acute Physiology and Chronic Health Evaluation II, *ICU* intensive care unit, *LOS* length of stay, *PSQI* Pittsburgh Sleep Quality Index

*p < 0.05

### Disability at follow-up

**Fig. 2:**
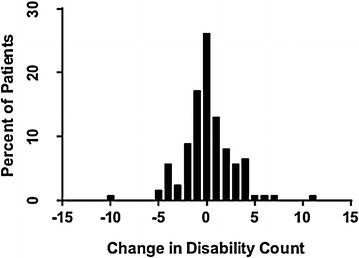
Change in disability counts from ICU admission to follow-up for 122 patients completing BADL/IADL instruments. *Legend* For changes in disability counts, positive values indicate increased disability at follow-up. *BADL/IADL* basic and instrumental activities of daily living, *ICU* intensive care unit

Figure 2 shows the distribution of disability scores for the 122 completing BADL/IADL instruments, measured as the change in disability from baseline to follow-up. Overall, the prevalence of worsening disability at follow-up relative to ICU admission was 37% (*n* = 45). The mean change in disability scores for the cohort was 0.15 (SD 2.62). Of the 77 patients who had stable or improved disability scores from baseline to follow-up, 26% (*n* = 20) had delirium during ICU stay. By contrast, among patients who showed worsening in their disability, 51% (*n* = 23) were delirium positive in the ICU.

In the linear regression model of change in disability, lower baseline disability scores (*p* < 0.001), older age (*p* = 0.048), and less time to follow-up interview (*p* = 0.024) were significantly associated with having worsening follow-up disability scores (Table 3). Total days of delirium, APACHE II scores, baseline depression, hospital length of stay, and mechanical ventilation status were not significantly associated with change in disability. Notably, when delirium was coded as a binary variable, its association with change in disability showed a trend toward statistical significance with a point estimate of 0.903 (95% confidence interval (− 0.090, 1.896), *p* = 0.077). The results of other explanatory variables in the latter model were unchanged. Finally, in a model including only patients with persistent delirium (i.e., those with at least 2 days of delirium, *n* = 32), only baseline disability scores were associated with worse disability scores at follow-up (data not shown).

**Table 3:** Linear regression analysis for factors associated with change in disability scores from baseline to follow-up (*n* = 122)

Factor	Point estimate (95% confidence interval)	*p* value
Age	0.030 (0.001, 0.059)	0.048*
Female gender	0.015 (− 0.837, 0.868)	0.972
Baseline disability	− 0.267 (− 0.385, − 0.148)	< 0.001*
Preexisting depression	− 0.330 (− 1.401, 0.742)	0.548
APACHE II	0.027 (− 0.064, 0.119)	0.562
Hospital LOS	0.038 (− 0.013, 0.089)	0.149
Mechanically ventilated during ICU admission	0.240 (− 1.042, 1.523)	0.714
Time to interview in days	− 0.008 (− 0.014, − 0.001)	0.024*
Total days of delirium	0.213 (− 0.141, 0.568)	0.240

*APACHE II* Acute Physiology and Chronic Health Evaluation II, *ICU* intensive care unit, *LOS* length of stay, *PSQI* Pittsburgh Sleep Quality Index

*p < 0.05

## Discussion

Our study in a cohort of MICU survivors indicates that post-discharge impairment in sleep and functional disability is highly prevalent, with possible links to MICU factors such as delirium.

Subjective sleep disturbance was identified in 63% of post-discharge patients at an average of 4- to 5-month follow-up. This finding is consistent with other studies of post-ICU patients, in which the prevalence of sleep disturbance ranges from 22 to 57% from 3 to 6 months after discharge [12]. Furthermore, after adjusting for other important covariates, increasing total days of MICU delirium were significantly associated with increased post-discharge sleep disturbance—the observed rate ratio (1.11) suggests that each additional day of MICU delirium raises the PSQI score by 11%. While there is a hypothesized relationship between delirium and sleep disruption within the MICU [26], to our knowledge this is the first study to demonstrate delirium as a factor associated with poor sleep after critical illness. Given that delirium is a syndrome of acute brain dysfunction associated with long-term cognitive impairment, one hypothesis is that a similar persistent brain dysfunction increases sleep disruption in critical illness survivors [9, 37]. Overall, our results link a prevalent MICU neurocognitive condition to sleep disturbance in post-ICU patients.

Notably, regression analysis also identified that older age was associated with improved sleep scores at follow-up. This finding differs from several studies in post-ICU patients which found that older age was associated with worse sleep, albeit using different sleep instruments in heterogeneous critically ill populations [38–40]. Furthermore, in non-critically ill populations, age is consistently associated with decreased total sleep time and sleep efficiency, less time spent in deep sleep stages, and worse self-reported sleep [41]. Although older age has been associated with improved sleep quality in some studies [42], this finding may reflect a self-report bias, wherein older adults normalize conditions (in this case, worse sleep) to which they have been chronically accustomed.

This study also assessed functional status in critical illness survivors using a composite of BADLs and IADLs. Accounting for baseline disability just prior to MICU admission, 37% of patients reported worsening disability at follow-up. In our model, patients who were older and nearer to their time of discharge were more likely to show increased disability at follow-up. Furthermore, lower baseline disability scores were significantly associated with having increases in disability at follow-up. This latter result possibly reflects a ceiling effect, wherein patients who are less disabled at baseline have greater capacity for worsening of their post-discharge disability relative to more disabled patients.

While total days of MICU delirium were not significantly associated with increased functional disability at follow-up (*p* = 0.240), delirium as a categorical variable (i.e., the presence of ICU delirium for at least one day) showed a trend toward association with increases in disability at follow-up (*p* = 0.077). The prior literature to date has been equivocal in assessing delirium as a risk factor for disability after critical illness. In a 2017 systematic review of post-ICU IADL dependencies, two of four studies assessing delirium found a significant association between delirium and worse post-ICU IADL scores [22, 24, 27, 28]. Several studies have also been conflicting regarding the association between BADLs and ICU delirium [24, 27, 28, 43]. Given our modest sample size, and the trend toward significance when using a categorical indicator for delirium, our results suggest a potential association between delirium and change in disability.

Overall, post-ICU impairments such as sleep disturbance and functional disability have been hypothesized to contribute to the “post-ICU syndrome,” in which patients have decreased physiologic reserve following critical illness and are at higher risk of hospital readmission [44]. Furthermore, these poor patient-centered outcomes likely interact with post-ICU comorbidities such as depression, anxiety, and reduced quality of life [19, 45–47]. In our sample, post-ICU patients experienced a high prevalence of sleep disturbance and increased disability—the former being significantly associated with ICU delirium and the latter being marginally associated. These data reinforce the importance of both delirium prevention and the need to identify the patients most at risk of poor outcomes after surviving critical illness.

There are several limitations to this study. First, a sizeable number of potentially eligible patients were unable to be contacted (24%) or survived through discharge but died before follow-up (22%). As a result, our sample may not represent the sickest ICU survivors who are most likely to experience mortality within the first several months after discharge. Second, without baseline or within-ICU data on patient sleep, we cannot make conclusions regarding changes in sleep parameters that occur after critical illness admission and furthermore cannot ascertain whether baseline or in-ICU sleep disturbance accounted for delirium positivity in these patients. For example, patients with profound in-ICU sleep disturbance leading to acute confusion or disorientation may have met criteria for delirium either by our in-person or chart-based review methods, which could confound the observed relationship between ICU delirium and long-term sleep disturbance. Third, we did not have data on cognitive function post-ICU, which may be an important confounder in our results given that delirium and sleep are potentially linked to this variable. Patients that completed the follow-up PSQI, however, were subjectively deemed to have sufficient cognitive ability to communicate in the interview. Finally, while our modest sample size may have precluded the detection of an association between delirium and post-discharge disability, it comprises a respectably large sample relative to comparable studies of survivors of critical illness.

## Conclusions

Critical illness represents a severe physiologic stress with a high degree of mortality and morbidity. In a cohort of MICU survivors assessed in the year following discharge, poor sleep quality and increased disability were highly prevalent. After adjusting for important covariates, total days of MICU delirium were significantly associated with increased sleep disturbance, and the occurrence of delirium showed a trend toward association with increased disability. Overall, these results have important implications for identifying MICU patients that are likely to experience difficulty with sleep and function in the year following hospital discharge. MICU delirium is an especially important factor to consider in planning for post-discharge care of these patients.

## Abbreviations

APACHE II: Acute Physiology and Chronic Health Evaluation II
BADLs: basic activities of daily living
CAM-ICU: Confusion Assessment Method for the ICU
IADLs: instrumental activities of daily living
ICU: intensive care unit
MICU: medical intensive care unit
PSQI: Pittsburgh Sleep Quality Index
